# FANCD2 functions as a critical factor downstream of MiTF to maintain the proliferation and survival of melanoma cells

**DOI:** 10.1038/srep36539

**Published:** 2016-11-09

**Authors:** Julie Bourseguin, Caroline Bonet, Emilie Renaud, Charlotte Pandiani, Marina Boncompagni, Sandy Giuliano, Patrycja Pawlikowska, Houda Karmous-Benailly, Robert Ballotti, Filippo Rosselli, Corine Bertolotto

**Affiliations:** 1 CNRS UMR 8200, Gustave Roussy, Villejuif, F-94805 France; 2 Gustave Roussy, Université Paris-Saclay, Villejuif, F-94805, France; 3 Equipe Labellisée “ARC”, C3M, Nice, F-06204, France; 4 Inserm, U1065, Equipe 1, Biologie et pathologies des mélanocytes: de la pigmentation cutanée au mélanome, C3M, Nice, F-06204, France; 5 Université Nice Sophia-Antipolis, UFR Médecine, Nice, F-06107, France; 6 Service de génétique médicale, CHU de Nice - Hôpital l’Archet 2, Nice, F-06200, France

## Abstract

Proteins involved in genetic stability maintenance and safeguarding DNA replication act not only against cancer initiation but could also play a major role in sustaining cancer progression. Here, we report that the FANC pathway is highly expressed in metastatic melanoma harboring the oncogenic microphthalmia-associated transcription factor (MiTF). We show that MiTF downregulation in melanoma cells lowers the expression of several *FANC* genes and proteins. Moreover, we observe that, similarly to the consequence of MiTF downregulation, FANC pathway silencing alters proliferation, migration and senescence of human melanoma cells. We demonstrate that the FANC pathway acts downstream MiTF and establish the existence of an epistatic relationship between MiTF and the FANC pathway. Our findings point to a central role of the FANC pathway in cellular and chromosomal resistance to both DNA damage and targeted therapies in melanoma cells. Thus, the FANC pathway is a promising new therapeutic target in melanoma treatment.

In spite of recent clinical breakthroughs, cutaneous metastatic melanoma remains the most deadly form of skin cancer^1^. Extensive analysis previously reported that the mRNAs coding proteins involved in double-strand break repair and in the rescue of stalled DNA replication forks, including several members of the FANC pathway, are over-expressed in metastatic melanomas and are associated with poor patient survival^2^,^3^. Indeed, proteins and pathways involved in DNA damage response (DDR) act not only as a tumour barrier but can also be required for tumour cell survival, proliferation and invasion^4^,^5^. Thus, DDR proteins represent a promising pharmacological target for improving therapeutic responses in melanoma.

The FANC pathway comprises the FANCcore complex (which includes FANCA, B, C, E, F, G, L and M), the E2 FANCT/UBE2T, the ID2 complex (FANCD2 and FANCI), subunits of two endonuclease complexes (FANCP/SLX4, FANCQ/XPF) and proteins involved in homologous recombination (HR) (FANCJ/BRIP1, FANCO/RAD51C, FANCD1/BRCA2, FANCN/PALB2, FANCR/RAD51 and FANCS/BRCA1)^6^. Stalled replication forks trigger the FANCcore complex-mediated monoubiquitination of FANCD2 and FANCI and their recruitment to subnuclear foci, thereby enabling the ID2 complex to coordinate DNA repair and replication restart *via* HR^7^,^8^,^9^,^10^. Bi-allelic mutations in a *FANC* gene are causative of the chromosome breakage syndrome Fanconi anemia (FA), which puts patients at risk for acute myeloid leukaemia and head and neck cancer^11^. To the better of our knowledge, melanomas were never reported in FA.

Both the absence of melanoma in FA patients and the observed over-expression of mRNAs encoding FANC proteins in metastatic melanoma implicitly suggest that the FANC pathway may be required to support melanoma development. Thus, we decided to decipher if and how the FANC pathway participates in melanomagenesis. We show here that a fully proficient FANC pathway is a key determinant for melanomagenesis ensuring the high proliferation and migration capabilities that characterize melanomas overexpressing the transcription factor microphthalmia (MiTF), which is amplified and/or mutated in a subset of patients with malignant melanoma where it acts as an oncogene^12^,^13^,^14^,^15^.

## Results

### FANC pathway silencing alters proliferation, migration, senescence and intracellular redox status of human melanoma cells

**Figure 1:**
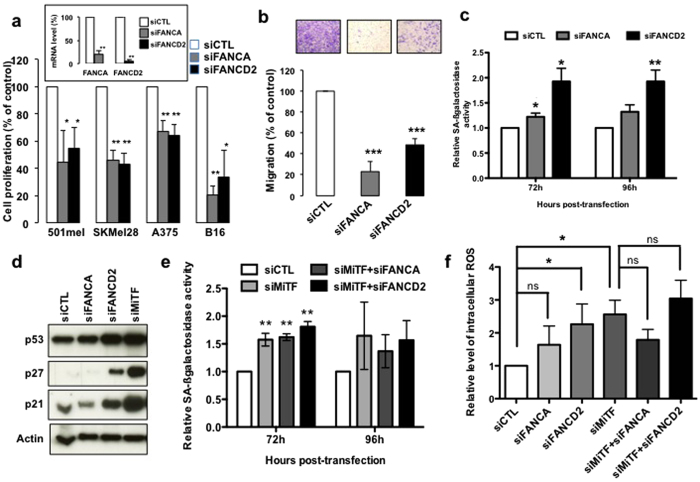
(**a**) Cell proliferation analysis as evaluated 4 days after transfection of the indicated human melanoma cell lines with control (CTL), FANCA or FANCD2 siRNA. The efficiency of the siRNA transfection in 501 mel melanoma cells is reported in the inset. The data are the means of 3 independent experiments. The error bars indicate S.D., ** and * indicate a statistically significant difference of p < 0.01 and p < 0.05, respectively. (**b**) Cell migration was assessed using a Boyden chamber assay. 501 mel cells were seeded on the upper trans-well chamber, and complete DMEM was added to those in the lower chamber. The cells that had adhered to the underside of the filters were fixed with 4% PFA, stained with 0.4% crystal violet and 3 random fields were counted at 20x magnification using the NIH-ImageJ analysis software. The values represent the means + S.D. of three independent experiments; *** indicate a statistically significant difference of p < 0.001. Representative images are shown. (**c**) SA-βGal relative activity evaluated via FACS analysis 72 or 96 h after transfection with the indicated siRNA in 501 mel melanoma cells. The data are the means of 3 independent experiments. The error bars indicate S.D. Statistical analysis was performed using Student’s T test, * indicates p < 0.05 and ** indicates p < 0.01. (**d**) Immunoblot showing the effects of FANCA, FANCD2 or MiTF silencing in 501 mel melanoma cells 96 h after siRNA transfection. The expression of p53, p21 and p27 was assessed. Actin expression was used as the loading control. (**e**) SA-βGal relative activity evaluated via FACS analysis 72 or 96 h after transfection with the indicated siRNA in 501 mel melanoma cells. The data are the means of 3 independent experiments. Statistical analysis was performed using Student’s T test. ** indicates p < 0.01 in relation to siCTL transfected cells. (**f**) Relative intracellular reactive oxygen species (ROS) measured with the DCF-DA probe via flow cytometry in 501 mel cells 96 h after transfection with the indicated siRNA. The data are the means of 3 independent experiments. The error bars indicate S.D. Statistical analysis was performed using Student’s T test, * indicates p < 0.05.

To comprehensively evaluate the role of the FANC pathway in melanoma behaviour, we silenced in several human melanoma cells either FANCA, the most frequently mutated *FANC* gene in FA syndrome, or FANCD2, the monoubiquitination and subnuclear relocalisation of which constitute a readout for the activation of the pathway. FANCA or FANCD2 silencing induced a significant decrease in the number of proliferating cells (Fig. 1a) and in their colony formation ability ( Supplementary Fig. 1a). Moreover, FANCA or FANCD2 knockdown dramatically reduced the migration ability of the melanoma cells (Fig. 1b) and suppressed their capacity to sustain anchorage-independent cell growth (Supplementary Fig. 1b), which are indicators of tumorigenicity and invasiveness.

FANC proteins silencing resulted in increased cell size and granularity (Supplementary Fig. 1c,d), which are associated with cellular senescence. Specifically, by measuring senescence-associated β-galactosidase activity (SA-βGal) via fluorescence-activated cell sorting (FACS) analysis or blinded individual microscopic scoring, we consistently demonstrated that FANCD2 silencing specifically induced a significant increase in SA-βGal activity, in the expression of p53, p21 and p27, as well as intracellular reactive oxygen species (ROS) accumulation, which are known inducers and/or markers of the senescence process (Fig. 1c,d,e,f and Supplementary Fig. 1e,f,g)^16^,^17^,^18^. To rule out non-specific effect we assessed the effect of a second siRNA to FANCA (FANCA#2) and FANCD2 (FANCD2#2). As previously observed, these siRNA, which efficiency is shown (Supplementary Figure 1h), reduced cell proliferation as illustrated by a reduced number of cells (Supplementary Figure 1i) and increased expression of cell cycle inhibitors and promoted senescence phenotypes such as increase in SA-βGal activity and increase in 53BP1 foci (Supplementary Fig. 1j,k). Thus, FANC pathway silencing in melanoma cells resulted in growth arrest, migration defect and senescence, which are phenotypes that we previously observed following the silencing of the oncogenic microphthalmia-associated transcription factor (MiTF)^19^. We therefore hypothesised a potential interplay between the MiTF and the FANC pathway. Accordingly, the simultaneous silencing of FANCA or FANCD2 with MiTF resulted in the same effect following the silencing of MiTF alone in terms of ROS production and senescence (Fig. 1e,f).

### FANC pathway and/or MiTF silencing generate genetic instability and hypersensitivity to MMC-induced DNA damage

**Figure 2:**
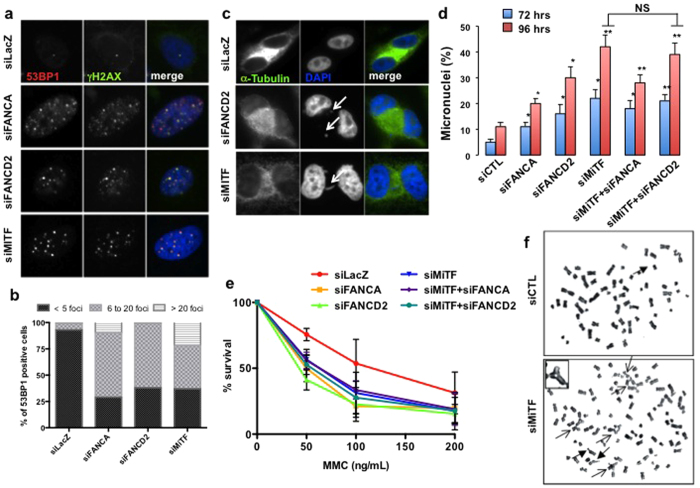
(**a**) Photomicrographs of 501 mel cells analysed 72 h after transfection with the indicated siRNA showing the level of 53BP1 (Red) and γH2AX (Green) subnuclear foci. DNA was stained via DAPI. (**b**) Histograms showing the percentage of 53BP1-positive cells classified as a function of the level of foci per cell. The data shown are from one representative experiment. (**c**) Photomicrographs of 501 mel cells captured 72 h after transfection with the indicated siRNA showing examples of analysed mitotic abnormalities, specifically micronuclei and anaphase bridges, indicated by arrows. Cells were treated with 10 ng/ml of MMC and 2 μg/ml of Cytochalasin B over-night before fixation. (**d**) Histograms showing the percentage of 501 mel cells presenting micronuclei at 72 or 96 h after transfection with the indicated siRNA. The data are the means of 3 independent experiments. The error bars indicate S.D. The statistical analysis was performed using Student’s T test. * indicates p < 0.05 and ** indicates p < 0.01. N.S., Not Significant. (**e**) Survival analysis in 501 mel cells exposed to increasing doses of mitomycin C. Each point represents the means of 3 independent experiments. The error bars indicate S.D. (**f**) 501 mel cells were transfected with control or MiTF-specific siRNA; 96 h later, these cells were exposed to mitomycin C (0.01 μg/ml) for 24 h. Metaphase spreads were analysed for chromosome breaks and radial figures. Representative images of radial figures and chromosome breaks are shown. The arrow indicates chromosomal aberrations. The inset is a magnification of a tri-radial chromosome.

Similar to the consequences of FANC pathway loss-of-function, MiTF-silenced cells showed sustained recruitment of γH2AX and 53BP1 foci, increased frequency of chromosome aberrations, anaphase abnormalities (i.e., anaphase bridges and chromosome loss) and post-mitotic micronuclei, indicating that MiTF-depletion results in DNA breakage as possible consequence of an altered rescue of stalled replication forks (Fig. 2a,b,c,d and Supplementary Fig. 2a,b). Specifically, less than 10% of exponentially growing melanoma cells displayed more than five 53BP1 foci per nucleus, a percentage that exceeded 60% when MiTF, FANCA or FANCD2 were silenced individually (Fig. 2b and Supplementary Fig. 2). Moreover, MiTF-depleted cells presented a cellular and chromosomal hypersensitivity to ICL-inducing agents similar to that reported in FANCA- or FANCD2-depleted melanoma cells (Fig. 2e,f, Supplementary Fig. 2c,d,e and data not shown). The silencing of both MiTF and FANCA or FANCD2 did not show additive or synergistic effects in terms of micronuclei induction or cellular sensitivity to ICLs (Fig. 2d,e). A more elevated level of cell death, a more pronounced growth arrest or both could be responsible for the slightly reduced level of micronucleated cells observed 96 hours post-transfection in the MITF-FANCA double-depleted cells compared to the MITF-depleted cells.

Finally, by flow cytometry we quantified cell cycle distribution in BrdU and/or propidium iodide stained cell populations (Supplementary Fig. 2f,g), demonstrating that, in melanoma cells, FANC and MiTF depletion with specific siRNAs triggers a similar G0/G1 phase accumulation together with a corresponding reduction in the frequency of S-phase cells, as evaluated 72 hours post-siRNA transfection.

Collectively, our data support the hypothesis that MiTF and the FANC proteins operate within one pathway to ensure DNA integrity, high proliferation and survival in melanoma.

### MiTF is involved in FANC genes expression

**Figure 3:**
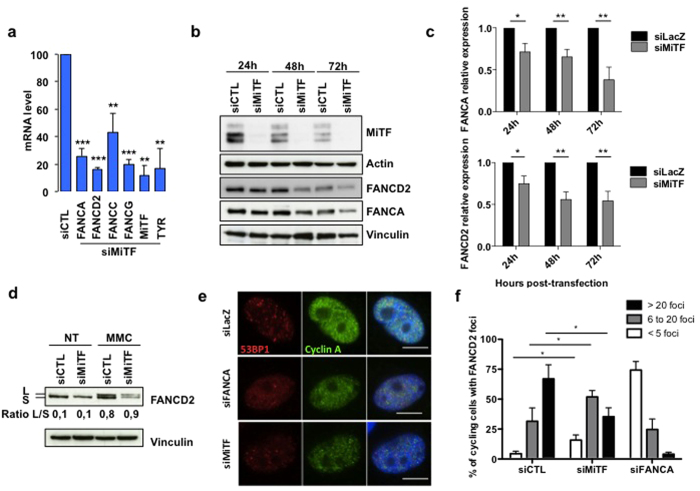
(**a**) Histograms showing the relative level of the indicated mRNA 48 h after transfection with siRNA targeting MiTF expression in 501 mel. The specific MiTF target tyrosinase (Tyr) was used as a control for the loss-of-function of the transcription factor. The data are the means of 3 independent experiments. Statistical analysis was performed using Student’s T test, ** indicates p < 0.01 and *** indicates p < 0.001. (**b**) Immunoblot showing the time-dependent consequences of the siRNA-mediated downregulation of MiTF on FANCD2 and FANCA levels. MiTF expression also decreased spontaneously as a consequence of culture time; FANCA and FANCD2 decreased accordingly. Actin and Vinculin were used as loading controls. (**c**) Histograms showing the quantification of the relative FANCA (top) or FANCD2 (bottom) levels in siMiTF-depleted 501 mel cells. Data are the means of three independent experiments. The error bars indicate S.D. Statistical analysis was performed using Student’s T test, * indicates p < 0.05 and ** indicates p < 0.01. (**d**) Immunoblot showing DNA damage-induced FANCD2 monoubiquitination in 501 mel cells that were untreated (NT) or exposed to MMC (1 μg/ml 1 h) 48 h after transfection with the indicated siRNA. Vinculin was used as loading control. The L/S ratio was calculated using ImageJ software. (**e**) Photomicrographs showing examples of FANCD2 subnuclear foci (red) in S-G2 phases cells, i.e., positively stained with Cyclin A (green). DNA was stained with DAPI. The cells were treated with MMC (1 μg/ml 1 h) 72 h after transfection with the indicated siRNA. (**f**) Histograms showing the quantification of the data from experiments presented in (**e**). The data are the means of 3 independent experiments. The statistical analysis was performed using Student’s T test, * indicates p < 0.05.

In line with a crosstalk between MiTF and the FANC pathway, MiTF silencing in melanoma cells triggered a strong reduction in the mRNA level of 4 analysed *FANC* genes: *FANCA, FANCD2, FANCC* and *FANCG* (Fig. 3a and Supplementary Fig. 3a). Using recently described MiTF chromatin immunoprecipitation sequencing (ChIP-seq) data set, we investigated the MiTF binding profile in the genomic region of *FANCD2* and *FANCA* (Supplementary Fig. 3b). We identified several significantly enriched MiTF-binding sites also containing E-box motifs, a known binding motif of MiTF^20^,^21^. Moreover, increased transcription correlates with the presence of H3K27ac modification. These data, together with reduced FANCD2 and FANCA mRNA level observed following MiTF-depletion, indicate a direct role for MiTF in the transcriptional activation of *FANCD2* and *FANCA* through binding in enhancer and promoter regions. We have indeed observed a modest activation of the transcriptional activity of the FANCD2 promoter by MiTF, consistently with the fact that MiTF binding sites are mainly localized in intronic regions (Supplementary Fig 3c). Contrastinlgy, we found no MITF binding in the genomic region of *FANCC* and *FANCG*, indicating an indirect regulation of these genes by MITF.

Likewise, immunoblot analysis demonstrated a robust and significant time-dependent decrease in the expression of both FANCA and FANCD2 following MiTF silencing (Fig. 3b,c, and Supplementary Fig. 3d). To rule out non-specific effects a second MiTF siRNA was used and showed similar inhibition of FANCA and FANCD2 expression (Supplementary Fig. 3e).

MMC exposure induced FANCD2 monoubiquitination and relocalisation to subnuclear foci where it largely colocalised with γH2AX foci (Fig. 3d,e,f). In MMC-treated MiTF-silenced cells, despite the reduced levels of FANCA and FANCD2, the latter underwent relatively normal monoubiquitination, but its monoubiquitinated form is able to aggregate into subnuclear foci in cyclin A-positive cells (i.e., cells into the S/G2 phase) (Fig. 3d,e,f). Obviously, as might be expected, the quantity of FANCD2 foci per cell is significantly reduced in MiTF-silenced cells (Fig. 3f).

Thus, MiTF silencing appears to alter the expression and capacity of the FANC pathway.

### FANC pathway is necessary to maintain genetic stability but dispensable to downregulate ROS levels downstream MiTF depletion

**Figure 4:**
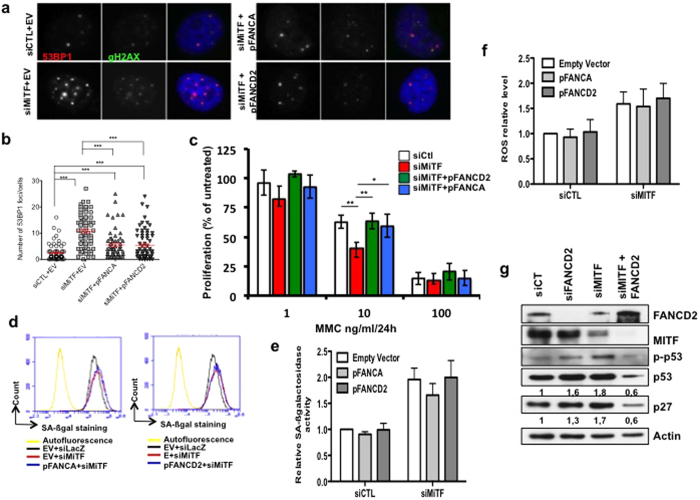
(**a**) Example of 501 mel cells transfected with the indicated siRNA and stained with anti-53BP1 (red) or γH2AX (green) to show the presence of subnuclear foci. DNA was stained with DAPI. (**b**) Quantification of the number of 53BP1 foci per nucleus. At least 75 cells from 10 different microscope fields were scored. Data are from one of three independent experiments with similar results. The statistical analysis was performed using Student’s T test, *** indicates p < 0.001. The mean and the S.E.M. are indicated in red. (**c**) Cell proliferation analysis of MITF-depleted or MITF-depleted and FANCD2 or FANCA overexpressing 501 mel melanoma cells exposed to increasing doses of mitomycin C. Each point represents the means of 3 independent experiments. Statistical analysis was performed using Student’s T test. * indicates p < 0.05 and ** indicates p < 0.01 in relation to siCTL or siMiTF-transfected cells. (**d**) SA-βGal relative activity evaluated via FACS analysis 72 h after transfection with the indicated plasmids and/or siRNA in 501 mel melanoma cells. (**e**) Relative SA-βGal activity in 501 mel cells 72 h after transfection with the indicated vectors and siRNA. The data are the means of at least 3 independent experiments. The error bars indicate S.D. (**f**) Relative intracellular ROS content in 501 mel cells 72 h after transfection with the indicated vectors and siRNA. The data are the means of at least 3 independent experiments. The error bars indicate S.D. (**g**) Immunoblot showing the effects of the transfection with the indicated vectors and siRNA in 501 mel melanoma cells on p53, P-p53, and p27 levels at 72 h post-transfection. Actin expression was used as a loading control.

To validate that increased DNA damage and cellular senescence in MiTF-silenced melanoma cells relied directly on the downregulation of the FANC pathway, we carried out experiments involving the forced expression of FANCA or FANCD2. The forced expression of FANCA or FANCD2 in MiTF-depleted cells significantly reduced the occurrence of spontaneous DNA damage, as indicated by γH2AX and 53BP1 foci formation (Fig. 4a,b) and foster melanoma cell proliferation in response to MMC exposure (Fig. 4c). On the contrary, neither FANCA nor FANCD2 forced expression is able to modify the siMiTF-associated increase in both β-Gal activity (Fig. 4d,e) and ROS intracellular levels, as determined by FACS analysis of DCFDA-stained cells (Fig. 4f). Since FANCD2 forced expression significantly reduced the increase in molecular markers associated to DNA damage response and growth arrest that are strongly expressed in siMiTF-depleted cells (Fig. 4g), we argue that FANCcore complex-mediated FANCD2 monoubiquitination appears to be required for an optimal DNA damage response in order to ensure genomic stability and cell proliferation but it is not sufficient *per se* for protecting cells against siMiTF-induced increased ROS content.

Thus, in MiTF depleted cells, senescence program follows also other pathways than the DNA-damage induced.

### FANC pathway participates to the poor outcome and vemurafenib resistance in melanoma

Collectively, our data suggest that the FANC pathway may participate in cancer progression and resistance to therapeutics and could be responsible for the poor outcome associated with melanoma.

**Figure 5:**
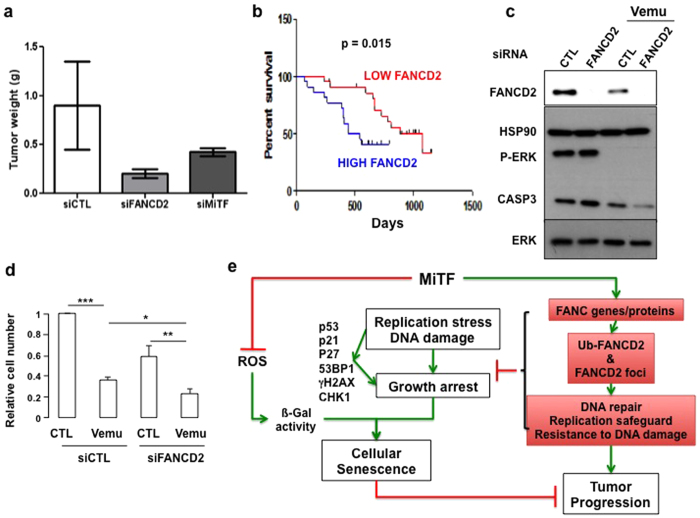
(**a**) Weight of the tumors developed in nude mice injected subcutaneously with 1 × 10^6^ B16 melanoma cells transfected with the indicated siRNA. Tumors were isolated 10 days after the injection, 3 mice for each siRNA were injected. (**b**) Survival curves for 44 metastatic melanoma patients were calculated using a Kaplan-Meier analysis with a Mantel-Cox long rank statistical significance test. Data are from the data set GSE19232^22^. (**c**) Immunoblot of control or FANCD2-suppressed WM9 melanoma cells that were untreated or exposed to vemurafenib (1 μM for 48 h) with the indicated antibodies. (**d**) Proliferation of WM9 or FANCD2-silenced WM9 melanoma cells that were untreated or exposed to vemurafenib (1 μM for 48 h). The statistical analysis was performed using Student’s T test, ** indicates p < 0.01 and *** indicates p < 0.001. (**e**) Schematic model summarising the observations made in this manuscript. FANC pathway, contributes to DNA damage response downstream of MiTF, avoiding the DNA damage-dependent senescence programme to take place, whereas MITF controls both ROS levels as well as DNA damage response, in a FANC pathway-independent and -dependent manner, respectively.

We further assessed the importance of a proficient FANC pathway for tumor growth *in vivo*. We injected subcutaneously nude mice with 1 × 10^6^ B16-F10 cells 72 hours after their transfection with siRNA targeting Fancd2 or Mitf, which efficiency is shown by Q-RT-PCR analysis (Supplementary Fig. 4a). As previously observed for human melanoma cell lines, 3 days after transfection both MiTF and Fancd2 siRNA reduced their corresponding protein expression level and MiTF siRNA in B16-F10 triggered a G0/G1 cell cycle arrest (Supplementary Fig. 2f and 4b). MiTF siRNA also decreased Fancd2 level (Supplementary Fig. 4b). Ten days later, mice were sacrificed, tumor mass isolated and weighted to estimate tumor growth capability. As reported in Fig. 5a, FANCD2 depletion strongly affects tumor development *in vivo*, clearly supporting the important role of a proficient FANC pathway in tumor progression and growth. Moreover, an analysis of datasets from patients with stage III and IV metastatic melanoma^22^ revealed that high FANCD2 expression significantly correlated with poorer prognosis (Fig. 5b, supplementary Fig. 4c). BRAF^V600E^ mutation, which leads to MAPK/ERK signalling pathway activation and hyperproliferation, is the most common causative mutation in melanoma^23^. Vemurafenib, a BRAF^V600E^ inhibitor, has been recently shown to trigger transient melanoma regression; patients rapidly developed resistance and subsequently relapsed^24^. We wondered if FANCD2 could influence vemurafenib response in BRAF^V600E^ melanoma cells. Immunoblot analysis showed that vemurafenib efficiently reduced ERK activation, decreased FANCD2 expression, as well as caused the disappearance of the zymogenic form of caspase 3, which occurred in association with cell growth arrest and death (Fig. 5c). The decrease in FANCD2 following vemurafenib treatment was validated by analysing a publicly available dataset (Supplementary Fig. 4d). More importantly, FANCD2 silencing improved and FANCD2 overexpression abolished the cellular effects of vemurafenib, demonstrating that FANCD2 has a protective function against the pharmacological effect of the vemurafenib (Fig. 5d and Supplementary Fig. 4e,f).

Altogether, our results demonstrate that FANCD2 participates in the cellular resistance of melanoma cells even to non-DNA damage therapeutics. Consequently, since the FANC pathway appears to oppose the efficiency of targeted therapies, specific inhibitors of FANCD2 activity/activation or inducers of its degradation could be useful therapeutic agents for increasing the efficiency of current melanoma treatments.

## Discussion

We previously showed through high throuput screening that MITF directly regulates a set of genes involved in DNA replication and repair^25^. However, it is not known whether these genes have a role in melanoma. We report here that the FANC pathway plays an unexpected yet key role in the proliferation and survival of melanoma cells downstream of MiTF (Fig. 5e). We identified MiTF as a critical regulator of the FANC pathway in melanoma and demonstrated that MiTF-silenced cells display the primary characteristics of FA cells, i.e., the cellular and chromosomal hypersensitivity to ICL-inducing agents. Our observations strongly support that thanks to its ability to cope with DNA lesions and replication stress the FANC pathway appears to be important for maintaining the high proliferative potential of melanoma cells as well as their high resistance to therapeutics.

It is noteworthy that silencing of FANCD2 in melanoma cells recapitulates all the key cellular phenotypes of FA cells while FANCA depletion fails to induce a significant increase in the intracellular level of ROS (Fig. 1f). This apparent discrepancy could be due to the fact that FANCA depletion by siRNA is, in general, not so strong as that obtained for FANCD2. Alternatively, the effect of FANCA deficiency in melanoma cells could have a less severe effect in term of senescence and ROS production than FANCD2 depletion. Indeed, all the analyzed markers of senescence (Fig. 1d) are less activated following FANCA depletion than following FANCD2 or MiTF downregulation.

Nevertheless, even if FANCD2 depletion resulted *per se* in an increasing intracellular level of ROS, MiTF downregulates ROS level in a FANC-pathway independent manner (Fig. 4f). Thus, as depicted in Fig. 5e, FANC pathway, contributes to DNA damage response downstream of MiTF, avoiding the DNA damage-dependent senescence programme to take place, whereas MITF controls both ROS levels as well as DNA damage response, in a FANC pathway-independent and -dependent manner, respectively.

It interesting to note that our data indicate that the FANC pathway influences also melanoma cells migration and invasiveness. Probably, both migration and invasiveness are independent from the DDR activity of the pathway, but, more realistically, linked to the recently reported FANCA loss-of-function-associated defect in CXCR5 neddylation, which decreases the membrane localisation of this receptor involved in cell motility, invasion and homing^26^. Clearly, our data indicate that the FANC proteins have a central role in melanoma development, favouring growth, motility, invasiveness and DNA damage resistance of melanoma cells.

Although sustained MITF inhibition triggers the hallmarks of cellular senescence^27^, MiTF expression must transiently decrease to enable melanoma cell migration and invasion but high MiTF level must be recovered to allow the growth of the metastases^13^,^28^. This oscillatory regulation of MiTF expression is a critical determinant of melanoma metastasis development and could account for melanoma heterogeneity, plasticity and drug resistance. As FANCD2 expression was higher in melanoma cells with a proliferative signature compared to melanoma cells with an invasive signature ( http://www.jurmo.ch/hopp/hopp_mpse.php), FANCD2, within the MiTF/FANC axis might follow the same oscillatory model. Hence, the low MiTF phenotype-associated reduction in FANC expression during the metastatic process could contribute to increased genomic instability and the selection of cells that could seed and survive during the migration process. The cells would subsequently need to re-express MiTF and the FANC pathway once they reach their final destination, thereby contributing to the “genetic stability” and proliferation of the cells at the metastatic site.

DNA repair process has been shown to mediate resistance of cells to DNA-damage-induced cell death^29^. Whether they mediate the resistance of metastatic melanoma to chemo- and radio-therapy remained to be demonstrated. Here we show that reduction in FANCD2 improves the effect of vemurafenib. Vemurafenib has been shown to cause a slowdown in replication rate in melanoma cells. FANC pathway is required to cope with replication stressess preventing the accumulation of replication-associated DNA double strands breaks. Since vemurafenib exposure decreases FANC protein expression (Fig. 5c and Supplementary S4d), it is likely that vemurafenib treated cells have a reduced ability to cope with DNA damage and stalled replication forks. It is why the forced expression of FANCD2 allows the cells to better sustain proliferation: thanks to FANCD2 they are able to better manage replication difficulties. Thus, our observations support the idea that inhibition of DNA repair processes may have a therapeutic value in metastatic melanoma. In this context, some pharmacological inhibitors of FANCD2 have been recently identified. Although not really specific for FANCD2, their utilization as therapeutic agents could open new opportunities to increase the efficiency of melanomas treatments.

## Methods

### Cell culture

Human (501 mel, SKMel28, SKMel5 and WM9) and mouse (B16) melanoma cells were grown in DMEM supplemented with 7% FCS and 100 units/mL penicillin/50 μg/mL streptomycin at 37 °C in a humidified atmosphere containing 5% CO_2_.

### Transfection

Briefly, a single pulse of 20 nmol/L of small interfering RNA (siRNA) was administered to the cells at 50–70% confluency via transfection with 8 μL INTERFERin (Polyplus) in Opti-MEM for the time indicated in the figure legends. FANCA siRNAs were Smartpools (Dharmacon) and from Invitrogen (FANCA#2) 5′-GGGUCAAGAGGGAAAAAUATT-3′. MiTF siRNAs were from Invitrogen: 5′-GGUGAAUCGGAUCAUCAAG-3′ (siMiTF1) or 5′-AGCAGUACCUUUCUACCACTT-3′(siMiTF2). Human FANCD2 was targeted with two individual siRNA from Invitrogen 5′-GGAGAUUGAUGGUCUACUA-3′ and 5′-AACAGCCAUGGAUACACUUGATT-3′. Mouse Fancd2 was targeted with the Smartpool siRNA from Dharmacon. In the case of double transfection, the same total amount of siRNA was used with a control siRNA added to the single siRNA of interest.

pCDNA3 (empty vector), pFLAG-FANCD2 and pFLAG-FANCA were a gift of A. Constantinou’s lab (Montpellier). Briefly, 1 μg of plasmid was transfected to the cells using 6 μL JetPEI (Polyplus) in 150 mM NaCl for the time indicated in the figure legends. The medium was changed 6 h after transfection. For siRNA and plasmid cotransfection, the siRNA were transfected 8 h after seeding; the plasmids were transfected 16 h later.

### Treatment

As indicated in the figure legends, cells were treated with mitomycin C (MMC) 48 h after transfection at the concentration of 1 μg/mL for 1 h or 100 ng/mL for 24 h. After several washes, they were then incubated in fresh medium prior to collection for Western blot or fixation for immunofluorescence.

For micronuclei and anaphase bridges formation, cells were treated with 2 μg/mL of cytochalasin B (Sigma) with or without 50 ng/mL of MMC for 24 h.

WM9 cells were transfected with 50 nM siRNA or infected with adenovirus at at multiplicity of infection (MOI) of 15 for 48 h prior exposure to vemurafenib 1 mM for 48 h.

### RT-qPCR

qPCR was performed as previously reported^30^. The primer sequences for each cDNA were designed using qPrimerDepot ( http://primerdepot.nci.nih.gov) and are available upon request.

### ROS detection

Cells (50.10^3^) in 6-well dishes were transfected with the different siRNA and were incubated with 10 μM DCF-DA (Molecular Probes) for 30 min. The cells were washed with PBS, detached using HQ-tase (Perbio Science) and analysed via FACS on the green channel.

### Western blot

The collected cells were disrupted in lysis buffer [50 mM Tris-HCl pH7.9, 40 mM NaCl, 1 mM MgCl2, 0.1% SDS and 1% Benzonase (Novus) supplemented with protease and phosphatase inhibitors (Roche)]. After incubation for 15 min at room temperature, the protein concentrations were determined using the Bradford assay (Bio-Rad), and the samples were combined with 4X Laemmli buffer containing β-mercaptoethanol and denatured via boiling. The proteins (25 μg) were separated using SDS-PAGE, transferred onto a nitrocellulose membrane, and then exposed to the appropriate antibodies. The proteins were visualised using an enhanced chemiluminescence system. All western blot quantifications were performed using densitometry measures and the ImageJ software.

### Immunofluorescence

Cells grown on glass coverslips were fixed in 4% formaldehyde supplemented with 0.1% Triton X-100 for 15 min at room temperature prior to permeabilisation in 0.5% Triton X-100 for 5 min. After blocking with 3% BSA in PBS containing 0.05% Tween 20, the cells were stained for 1 h in blocking solution with antibodies. Primary antibody detection was achieved via incubation with anti-rabbit or anti-mouse Alexa Fluor 594- or 488-conjugated secondary antibodies (Invitrogen) for 45 min at room temperature. The slides were mounted in DAKO mounting medium supplemented with DAPI (Sigma) and examined at a magnification of 63× via fluorescence microscopy (Zeiss Axio Observer Z1). The images were captured with an ORCA-ER camera (Hamamatsu). The microscope and camera parameters were adjusted for each series of experiments to avoid signal saturation. Image processing and analysis were performed using ImageJ software.

### Antibodies

The following antibodies were used: mouse anti-MiTF (Abcam), mouse anti-FANCD2 (Santa Cruz), rabbit anti-FANCD2 (Abcam), rabbit anti-FANCA (Bethyl), mouse anti-Flag (Sigma), rabbit anti-p53 serine 15 (Cell Signaling Technology), mouse anti-p53 (Santa Cruz), rabbit anti p27 (Cell Signaling), rabbit anti-p21 (Santa Cruz), mouse anti-Cyclin-A (Abcam), rabbit anti-53BP1 (Abcam), mouse anti-gH2AX (Millipore), mouse anti-alpha-tubulin (Abcam), mouse anti-vinculin (Abcam), and goat anti-actin (Abcam).

### Chromosomal analysis (conventional karyotyping)

501 mel cells were transfected with control or MiTF-specific siRNA; 96 h later, these cells were exposed to DEB (0.01 μg/ml) for 48 h or mitomycin C (0.01 μg/ml) for 24 h. Briefly, the cells were arrested at metaphase by adding colchicine (1 μg/ml) to the growing culture for 6 h. The arrested cells were then collected via trypsinization with immediate neutralisation with cell culture medium followed by 5 min of centrifugation at 400 g. We incubated the cell pellet suspension in hypotonic buffer, potassium chloride (5.59 g/l in double distilled water), and sodium citrate (9.0 g/l in double-distilled water) at 1:1 (vol/vol) for 10 min at 37 °C. The cells were then pelleted at 400 g for 5 min to remove the hypotonic buffer and fixed with an ice-cold solution (methanol/acetic acid, 3:1, vol/vol). The fixative was changed once, and then the cells were fixed overnight at 4 °C. To spread the metaphase of the fixed cells onto slides, we dropped the cell suspension from a height onto a chilled, precleaned SuperFrost Plus microscopic slide (Gerhard Menzel, Braunschweig, Germany) that was slightly sloped on a freezer block. Then, the slides were breathed on to enhance spreading and were mounted after drying. The metaphase chromosomes of the metaphase-arrested cells were identified and captured using an automated cytogenetic scanner workstation (MetaSystems) for analysis. Twenty metaphase spreads of distinctly separated chromosomes were analysed for chromosomal abnormality.

### Migration assay

Cell migration was assessed using a Boyden chamber with 8-μm pore filter inserts for 24-well plates (BD Bioscience). Human 501 mel cells melanoma cells were seedd on the upper trans-well chamber; 7% DMEM was placed in the lower chamber. After 24 h, the cells that had adhered to the underside of the filters were fixed with 4% PFA and stained with 0.4% crystal violet. Three random fields were counted at x20 magnification. The results represent the average of samples from three independent experiments.

### Proliferation assay

Cells were plated at 1 × 10^5^ per well of a six-well plate. The cells were transfected 24 h after seeding and grown for 48 h prior to MMC treatment. The cells were incubated for 72 h in medium supplemented with or without MMC and counted with trypan blue using a Neubauer haemocytometer.

### Agarose colony-forming assays

The cells (5.10^3^) were transfected with 50 nM siRNA for 48 h, suspended in 1 ml of 7% FBS DMEM medium containing 0.3% agarose and plated on a 0.6% agarose base in 6-well plates. The cells were then placed in a 37 °C and 5% CO_2_ incubator. The colonies of cells allowed to grow for 14 days were stained with 0.04% crystal violet-2% ethanol in PBS for 30 min. The colony formation assay was performed in duplicate.

### Senescence-associated β-galactosidase assay

#### Fluorescence-based assay

Senescence-associated β-galactosidase (SA-β-Gal) activity was assessed with a fluorescence-based method for analysis via flow-cytometry, as previously described^31^. In brief, the cells were incubated for 1 h with bafilomycin A1 (Sigma) to prevent lysosomal acidification and were subsequently incubated with 5-dodecanoylaminofluorescein di-β-D-galactopyranoside (C12FDG, Invitrogen), a fluorogenic substrate for β-galactosidase. Fluorescence was detected using an Accuri C6 Flow Cytometer (BD Biosciences). Relative SA-β-Gal activity was estimated using the Median Fluorescence Intensity (MFI) of the population. Non-labelled samples were used to determine the autofluorescence.

#### Chromogenic assay

The senescence β-galactosidase staining kit (Cell Signaling Technology) was used to histochemically detect β-galactosidase activity. SA-β-Gal activity results in a cytoplasmic blue staining that can be visualised using light microscopy. The percentage of the means and SDs were derived from counting 100 cells after 72 h and 96 h.

### Cell cycle analysis

Cells were stained with propidium iodide (40 μg/ml) containing ribonuclease A (10 μg/ml) and were analyzed using a fluorescence activated cell sorter (MACSQuant Analyzer) and MACSQuantify software.

5-Bromo-2-deoxyuridine (BrdU, BD Bioscience) was added to the culture media at a final concentration of 10 μM for 10 min. Pelleted cells were resuspended in 30 mM HCl/0.5 mg/mL pepsin. BrdU was immunodetected with a mouse anti-BrdU antibody (DAKO, clone Bu20a) and a fluorescein-conjugated donkey anti-mouse antibody (Life Technologies) and cells were stained with PI. Flow cytometry analyses were performed using a Fluorescence was detected using an Accuri C6 Flow Cytometer (BD Biosciences).

### Tumour assay *in vivo*

Animal experiments were carried out at the Animal Facilities of the Gustave Roussy Institute under the conditions established by the French ministry (87–848) and European Community (Directive 86/609/CCE) on the protection of animals used for scientific purposes. The project was officially approved by the Animal Experimentation Ethics Committee of the Gustave Roussy Institute (IGR) and registered under the N° 26 by the IGR Department of Research and conducted in accordance with French Laws and regulations. The animals were maintained in a temperature-controlled facility (22 °C) on a 12-h light/dark cycle and were given free access to standard laboratory chow. Equal amount of B16 melanoma cells (0.5.10^6^ cell per condition) transfected with control siRNA or siRNA targeting FANCD2 or MiTF for 72 h were injected subcutaneously in nude mice. Ten days later, mice were sacrificed and tumour isolated and weighted.

### Statistical Analysis

The data are presented as averages ± SD and were analysed via Student’s T test using PRISM software. A p-value of 0.05 (*p < 0.05) or less (**p < 0.01 and ***p < 0.001) was interpreted as indicating statistical significance when comparing the experimental and control groups.

## Additional Information

**How to cite this article**: Bourseguin, J. *et al.* FANCD2 functions as a critical factor downstream of MiTF to maintain the proliferation and survival of melanoma cells. *Sci. Rep.* **6**, 36539; doi: 10.1038/srep36539 (2016).

**Publisher’s note**: Springer Nature remains neutral with regard to jurisdictional claims in published maps and institutional affiliations.

## Supplementary Material

Supplementary Information

## References

[b1] Rughani M. G., Gupta A. & Middleton M. R. New treatment approaches in melanoma: current research and clinical prospects. Therapeutic advances in medical oncology 5, 73–80, doi: 10.1177/1758834012463260 (2013). 23323148 PMC3539276

[b2] Kauffmann A. *et al.* High expression of DNA repair pathways is associated with metastasis in melanoma patients. Oncogene 27, 565–573, doi: 10.1038/sj.onc.1210700 (2008). 17891185

[b3] Winnepenninckx V. *et al.* Gene expression profiling of primary cutaneous melanoma and clinical outcome. J Natl Cancer Inst 98, 472–482, doi: 10.1093/jnci/djj103 (2006). 16595783

[b4] Hosoya N. & Miyagawa K. Targeting DNA damage response in cancer therapy. Cancer science 105, 370–388, doi: 10.1111/cas.12366 (2014). 24484288 PMC4317796

[b5] Kinzler K. W. & Vogelstein B. Cancer-susceptibility genes. Gatekeepers and caretakers. Nature 386, 761, 763, doi: 10.1038/386761a0 (1997). 9126728

[b6] Bogliolo M. & Surralles J. Fanconi anemia: a model disease for studies on human genetics and advanced therapeutics. Curr Opin Genet Dev 33, 32–40, doi: 10.1016/j.gde.2015.07.002 (2015). 26254775

[b7] Constantinou A. Rescue of replication failure by Fanconi anaemia proteins. Chromosoma 121, 21–36, doi: 10.1007/s00412-011-0349-2 (2012). 22057367 PMC3260432

[b8] Naim V. & Rosselli F. The FANC pathway and BLM collaborate during mitosis to prevent micro-nucleation and chromosome abnormalities. Nat Cell Biol 11, 761–768, doi: 10.1038/ncb1883 (2009). 19465921

[b9] Walden H. & Deans A. J. The Fanconi anemia DNA repair pathway: structural and functional insights into a complex disorder. Annual review of biophysics 43, 257–278, doi: 10.1146/annurev-biophys-051013-022737 (2014). 24773018

[b10] Wang L. C., Stone S., Hoatlin M. E. & Gautier J. Fanconi anemia proteins stabilize replication forks. DNA repair 7, 1973–1981, doi: 10.1016/j.dnarep.2008.08.005 (2008). 18786657 PMC2596863

[b11] Chirnomas S. D. & Kupfer G. M. The inherited bone marrow failure syndromes. Pediatric clinics of North America 60, 1291–1310, doi: 10.1016/j.pcl.2013.09.007 (2013). 24237972 PMC3875142

[b12] Bertolotto C. *et al.* A SUMOylation-defective MITF germline mutation predisposes to melanoma and renal carcinoma. Nature 480, 94–98, doi: 10.1038/nature10539 (2011). 22012259

[b13] Carreira S. *et al.* Mitf regulation of Dia1 controls melanoma proliferation and invasiveness. Genes Dev 20, 3426–3439, doi: 10.1101/gad.406406 (2006). 17182868 PMC1698449

[b14] Hartman M. L. & Czyz M. MITF in melanoma: mechanisms behind its expression and activity. Cellular and molecular life sciences: CMLS 72, 1249–1260, doi: 10.1007/s00018-014-1791-0 (2015). 25433395 PMC4363485

[b15] King R. *et al.* Microphthalmia transcription factor. A sensitive and specific melanocyte marker for MelanomaDiagnosis. Am J Pathol 155, 731–738 (1999). 10487831 10.1016/S0002-9440(10)65172-3 PMC1866880

[b16] Bringold F. & Serrano M. Tumor suppressors and oncogenes in cellular senescence. Experimental gerontology 35, 317–329 (2000). 10832053 10.1016/s0531-5565(00)00083-8

[b17] Campisi J. Aging, cellular senescence, and cancer. Annual review of physiology 75, 685–705, doi: 10.1146/annurev-physiol-030212-183653 (2013). PMC4166529 23140366

[b18] Carnero A. Markers of cellular senescence. Methods Mol Biol 965, 63–81, doi: 10.1007/978-1-62703-239-1_4 (2013). 23296651

[b19] Giuliano S., Ohanna M., Ballotti R. & Bertolotto C. Advances in melanoma senescence and potential clinical application. Pigment Cell Melanoma Res 24, 295–308, doi: 10.1111/j.1755-148X.2010.00820.x (2011). 21143770

[b20] Aksan I. & Goding C. R. Targeting the microphthalmia basic helix-loop-helix-leucine zipper transcription factor to a subset of E-box elements *in vitro* and *in vivo*. Mol Cell Biol 18, 6930–6938 (1998). 9819381 10.1128/mcb.18.12.6930 PMC109276

[b21] Cheli Y., Ohanna M., Ballotti R. & Bertolotto C. Fifteen-year quest for microphthalmia-associated transcription factor target genes. Pigment Cell Melanoma Res 23, 27–40, doi: 10.1111/j.1755-148X.2009.00653.x (2010). 19995375

[b22] Bogunovic D. *et al.* Immune profile and mitotic index of metastatic melanoma lesions enhance clinical staging in predicting patient survival. Proc Natl Acad Sci USA 106, 20429–20434, doi: 10.1073/pnas.0905139106 (2009). 19915147 PMC2787158

[b23] Bello D. M., Ariyan C. E. & Carvajal R. D. Melanoma mutagenesis and aberrant cell signaling. Cancer control: journal of the Moffitt Cancer Center 20, 261–281 (2013). 24077403 10.1177/107327481302000404

[b24] Garbe C., Abusaif S. & Eigentler T. K. Vemurafenib. Recent results in cancer research. Fortschritte der Krebsforschung. Progres dans les recherches sur le cancer 201, 215–225, doi: 10.1007/978-3-642-54490-3_13 (2014). 24756795

[b25] Strub T. *et al.* Essential role of microphthalmia transcription factor for DNA replication, mitosis and genomic stability in melanoma. Oncogene 30, 2319–2332, doi: 10.1038/onc.2010.612 (2011). 21258399

[b26] Renaudin X., Guervilly J. H., Aoufouchi S. & Rosselli F. Proteomic analysis reveals a FANCA-modulated neddylation pathway involved in CXCR5 membrane targeting and cell mobility. J Cell Sci 127, 3546–3554, doi: 10.1242/jcs.150706 (2014). 25015289

[b27] Giuliano S. *et al.* Microphthalmia-associated transcription factor controls the DNA damage response and a lineage-specific senescence program in melanomas. Cancer Res 70, 3813–3822 (2010). 20388797 10.1158/0008-5472.CAN-09-2913

[b28] Hoek K. S. *et al.* Novel MITF targets identified using a two-step DNA microarray strategy. Pigment Cell Melanoma Res 21, 665–676, doi: 10.1111/j.1755-148X.2008.00505.x (2008). 19067971

[b29] Sotiropoulou P. A. *et al.* Bcl-2 and accelerated DNA repair mediates resistance of hair follicle bulge stem cells to DNA-damage-induced cell death. Nat Cell Biol 12, 572–582, doi: 10.1038/ncb2059 (2010). 20473297

[b30] Ohanna M. *et al.* Senescent cells develop a PARP-1 and nuclear factor-{kappa}B-associated secretome (PNAS). Genes Dev 25, 1245–1261, doi: 10.1101/gad.625811 (2011). 21646373 PMC3127427

[b31] Debacq-Chainiaux F., Erusalimsky J. D., Campisi J. & Toussaint O. Protocols to detect senescence-associated beta-galactosidase (SA-betagal) activity, a biomarker of senescent cells in culture and *in vivo*. Nature protocols 4, 1798–1806, doi: 10.1038/nprot.2009.191 (2009). 20010931

