# Supplementary material for: FANCD2 functions as a critical factor downstream of MiTF to maintain the proliferation and survival of melanoma cells
Source: Sci Rep. 2016 Nov 9;6:36539. doi: 10.1038/srep36539 (PMC5101529; doi:10.1038/srep36539)
Supplement: Supplementary Information [file srep36539-s1.pdf]

FANCD2 functions as a critical factor downstream of MiTF to maintain the proliferation and survival of melanoma cells.

J. Bourseguin, C. Bonet, E. Renaud, C. Pandiani, M. Boncompagni, S. Giuliano, P. Pawlikowska, H. Karmous-Benailly, R. Ballotti, F. Rosselli, C. Bertolotto

Supplementary Figure Legends

Supplemental Figure 1:

a) Human melanoma cells were transfected with control (Ctl), FANCA or FANCD2 siRNA and allowed to grow for 14 days. The colonies were stained with 0.04% crystal violet 2% ethanol in PBS for 30 min. The colony formation assay was performed in duplicate. Representative images of colonies formed are shown.

b) Same as described in (a) but with cells grown in soft agar supplemented with 7% serum for 21 days.

c-d) Relative size (forward scatter) and granularity (side scatter) of 501mel cells were analysed using flow cytometry 96 h after transfection of the indicated siRNA. Mean values + SD are shown. Cell migration was assessed using a Boyden chamber assay. The values represent the means + SD of three independent experiments. The statistical analysis was performed using Student's T test, ** $p<0.01$, and *** $p<0.001$.

e) SA-βGal relative activity evaluated via FACS analysis 72 h after transfection with the indicated siRNA in 501mel melanoma cells.

f) SA-βGal activity evaluated via chromogenic assay in 501mel cells transfected with the indicated siRNA.

g) Quantification of the data obtained by chromogenic assay. The values represent

the means + SD of two independent experiments.

h) Immunoblot showing FANCA or FANCD2 expression upon FANCA#2 or FANCD2#2 knock-down for 96 hrs. ERK2 was used as a loading control.

i) Cell number count evaluated 96 hrs after transfection of 501mel human melanoma cell lines with control (CTL), FANCA#2 or FANCD2#2 siRNA. The error bars indicate S.D., *** and * indicate a statistically significant difference of $p<0.001$ and $p<0.05$, respectively.

j) SA-βGal activity evaluated via chromogenic assay in 501mel cells transfected with a second set of siRNA to FANCA or FANCD2 for 96h.

k) Photomicrographs of 501mel cells analysed 72 h after transfection with the indicated siRNA showing the level of 53BP1 (Red) subnuclear foci. DNA was stained via DAPI.

Supplemental Figure 2:

a) 501mel cells were transfected with the indicated siRNA for 72 hrs. Histograms showing the percentage of 53BP1-positive cells classified as a function of the level of foci per cell.

b) Photomicrographs of 501mel cells analysed 72 h after transfection with the indicated siRNA showing the level of 53BP1 (Red) and γH2AX (Green) subnuclear foci. DNA was stained via DAPI (left part). Quantification is shown on the right part.

c) 501mel cells were transfected with the indicated siRNA for 72 h. Cells were treated with 2µg/ml of Cytochalasin B over-night before fixation. The histograms show the percentage of cells with nucleocytoplasmic bridges.

d) 501mel cells were transfected with control or MiTF-specific siRNA; 96 hrs later,

these cells were exposed to DEB (0.01 μg/ml) for 48 h or mitomycin C (0.01 μg/ml) for 24 h. Metaphase spreads were analysed for chromosome breaks and radial figures. The numbers of metaphase with chromosome breaks and radial figures for each condition are shown.

e) Representative images of radial figures and chromosome breaks. The arrow indicates chromosomal aberrations.

f) FACS analysis of human (501mel and SkMel28) and mouse (B16) melanoma cells transfected with CTL, MiTF or FANCD2 siRNA for 72 hrs. Percentage of cell in the different phases of the cell cycle is shown.

g) Cells were examined for BrdU incorporation. Values represent the mean + SD.

Supplemental Figure 3:

a) Histogram showing the relative level of the indicated mRNA 48 h after transfection with control or MiTF siRNA in several human melanoma cell lines. The specific MiTF target, tyrosinase (Tyr), was used as control for the loss-of-function of the MiTF transcription factor. The results are the means are three independent experiments.

b) ChIP-seq profile showing significant MITF binding peaks in the *FANCA and FANCD2* genomic region (black arrowheads). The sequence under peak shows the presence of MITF-binding sites. The scale bar indicates the size of the genomic region in kilobases (kB). The H3K27ac track, associated with active transcription, is shown.

c) Luciferase assay using 501mel cells transfected with the FANCD2 promoter (Gene Copoiea) and with empty pcDNA3 vector or with vectors encoding wild type MITF. Forty eight hours post-transfection luciferase activity was assayed and normalized by the β-

galactosidase activity. Results are expressed as fold stimulation of the basal luciferase activity from cells transfected with empty vector. Data are means + SE of three experiments performed in triplicate.

d-e) Immunoblot showing the time-dependent consequences of the siRNA-mediated MiTF downregulation of FANCD2 and FANCA levels with two different MITF siRNA (siMiTF and siMiTF2). MiTF expression also spontaneously decreased as a consequence of the culture time; FANCA and FANCD2 decreased accordingly. HSP60 or ERK2 was used as a loading control.

Supplemental Figure 4:

a) B16 melanoma cells transfected with CTL, MiTF or FANCD2 siRNA for 48 hrs. Histogram showing the relative level of the indicated mRNA.

b) FACS analysis of B16 melanoma cells transfected with CTL, MiTF or FANCD2 siRNA for 48 hrs. Percentage of cell in the different phases of the cell cycle is shown. The statistical analysis was performed using Student's T test, *$p<0.05$, and ** $p<0.01$.

c) Survival curves for 162 metastatic melanoma patients were calculated using a Kaplan-Meier analysis with a Mantel-Cox long rank statistical significance test. PROGgeneV2 analysis (http://www.compbio.iupui.edu/proggene) of the dataset SKCM-TCGA (Goswami CP, Nakshatri H. PROGgene: gene expression based survival analysis web application for multiple cancers. *J Clin Bioinforma*. 2013 Oct 28;3(1):22. doi: 10.1186/2043-9113-3-22).

d) Box plots showing the expression of FANCD2 and FANCA in melanoma cells (GSE54711) that were untreated or exposed to vemurafenib (400 nM, 16 days).

e-f) Effect of FANCD2 overexpression (AdFD2) on 501mel or WM9 human

melanoma cell growth treated with 1 μM vemurafenib for 48 hrs. Western blot showing FANCD2 levels in infected cells, confirming the efficacy of both infection and expression as well as the efficacy of vemurafenib looking at the reduced level of p-ERK in treated cells. FANCD2 overexpression clearly impede the effects of vemurafenib on melanoma cells growth in both cellular models.

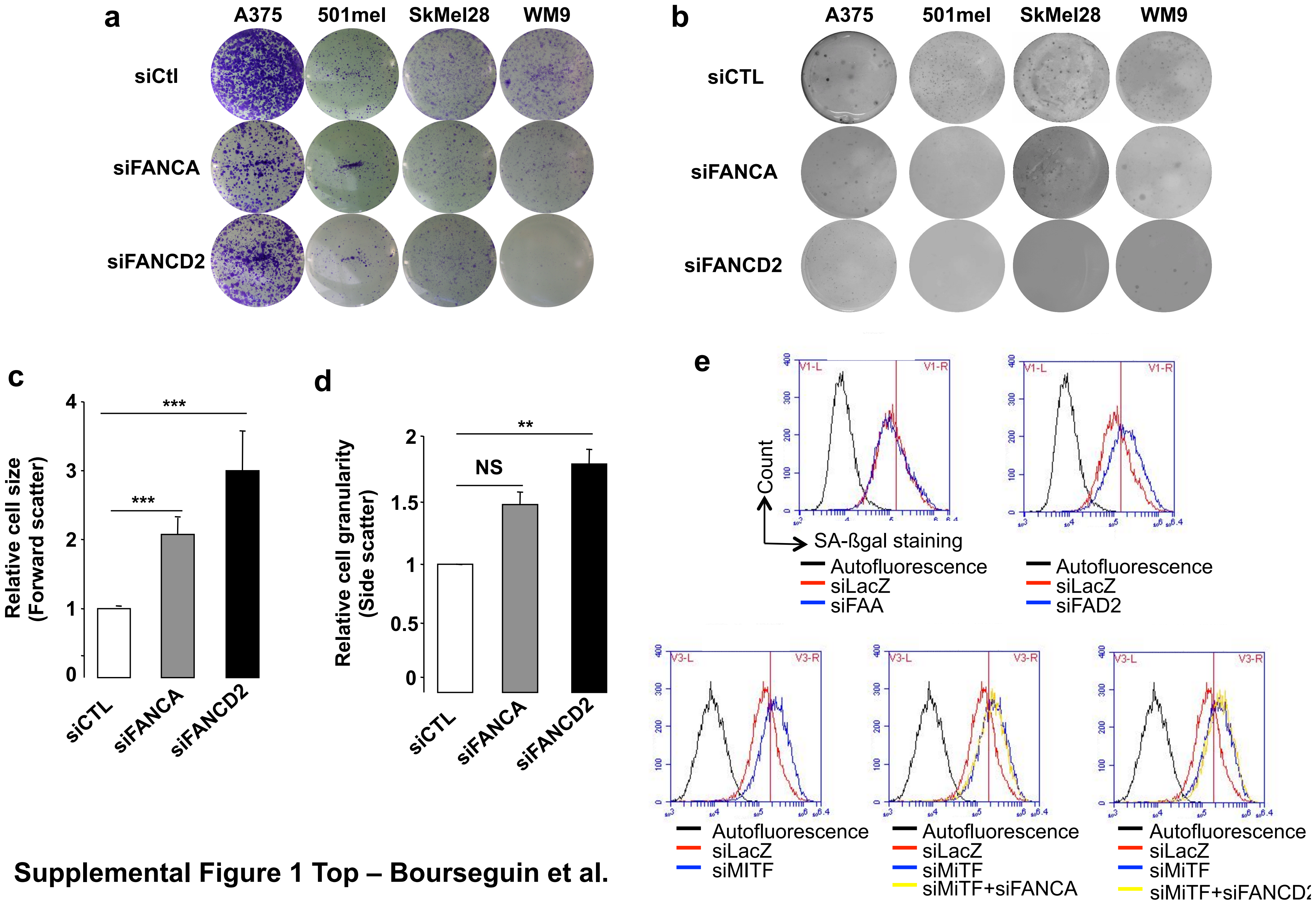

Supplemental Figure 1 Top – Bourseguin et al.

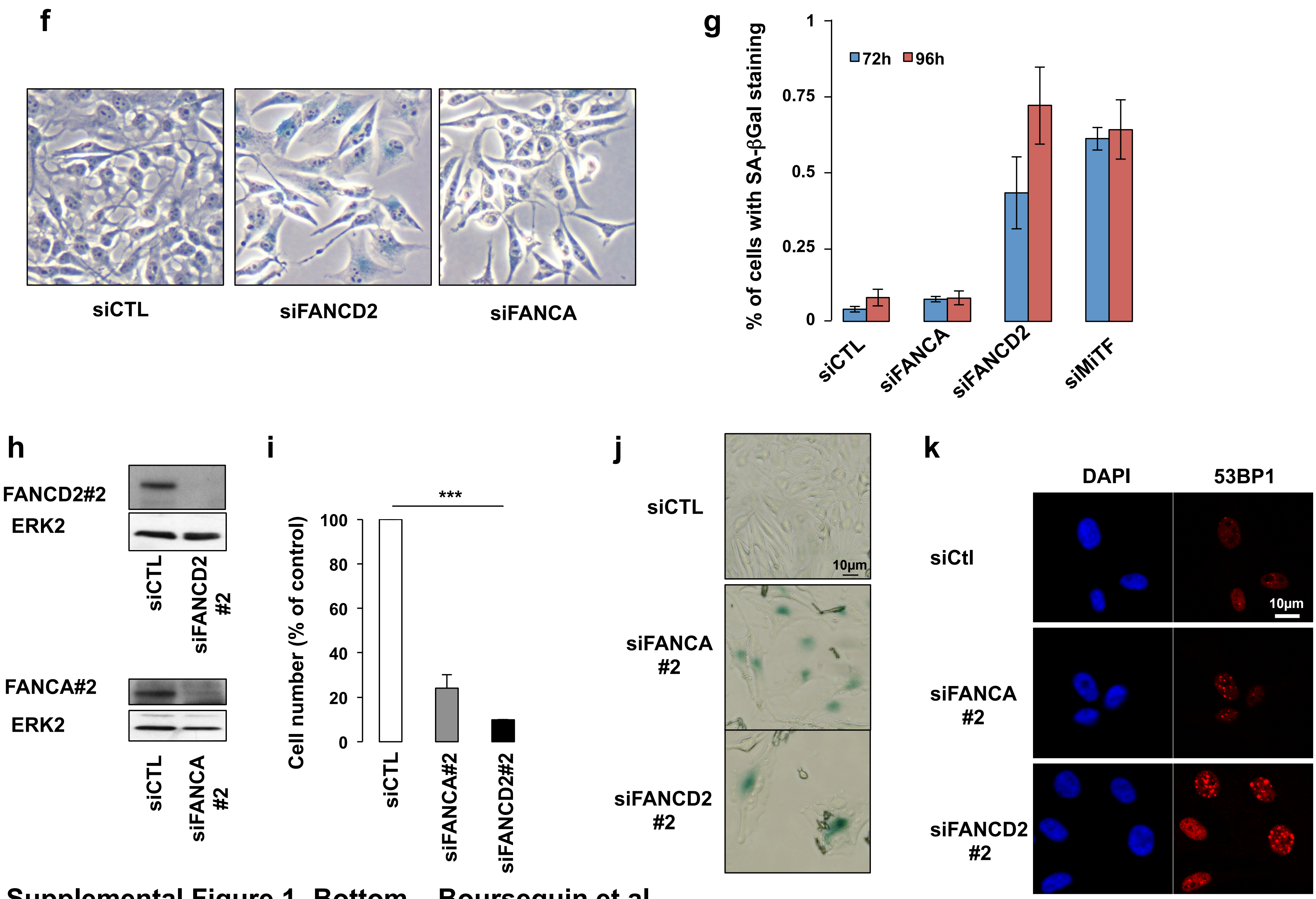

Supplemental Figure 1- Bottom – Bourseguin et al.

a

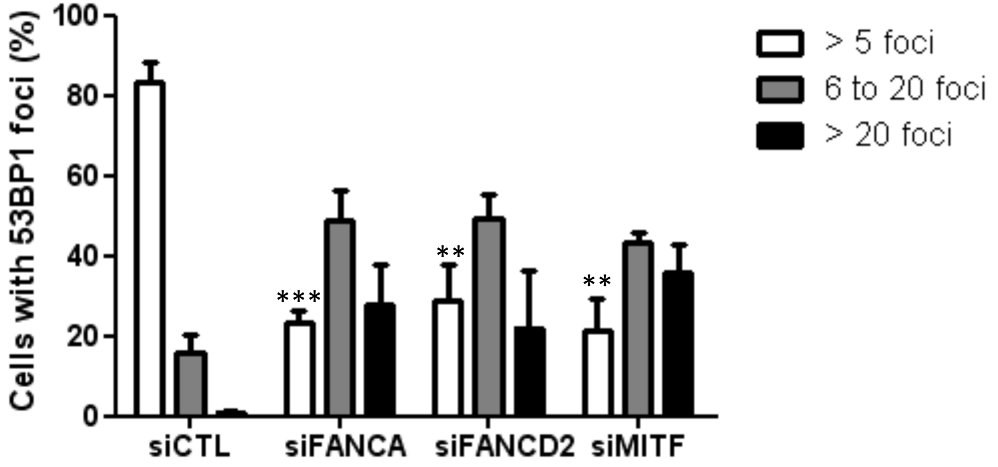

b

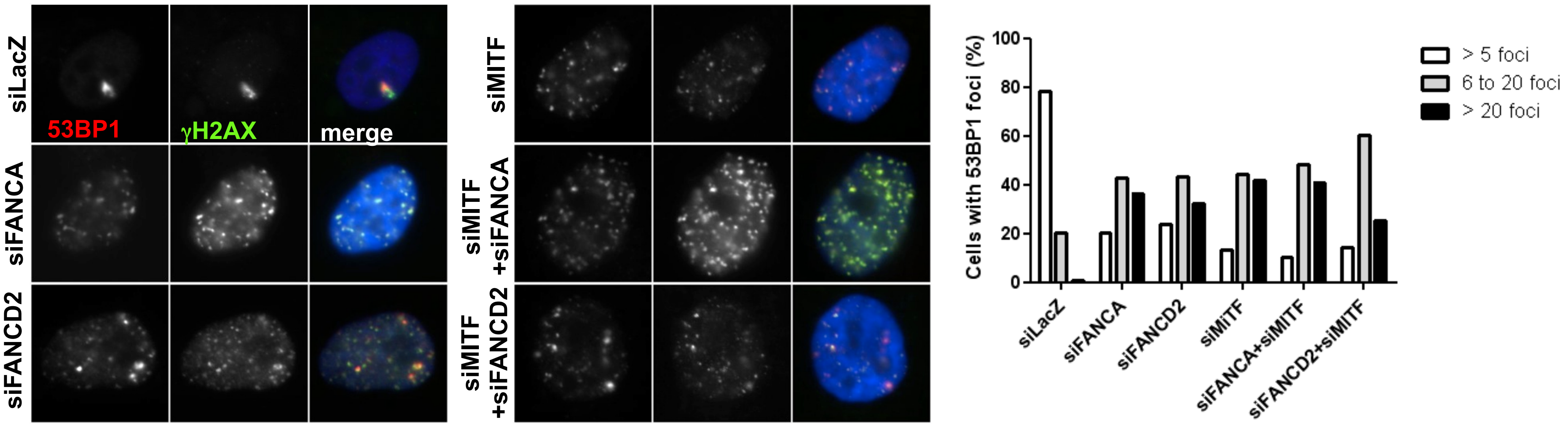

Supplemental Figure 2 Top – Bourseguin et al.

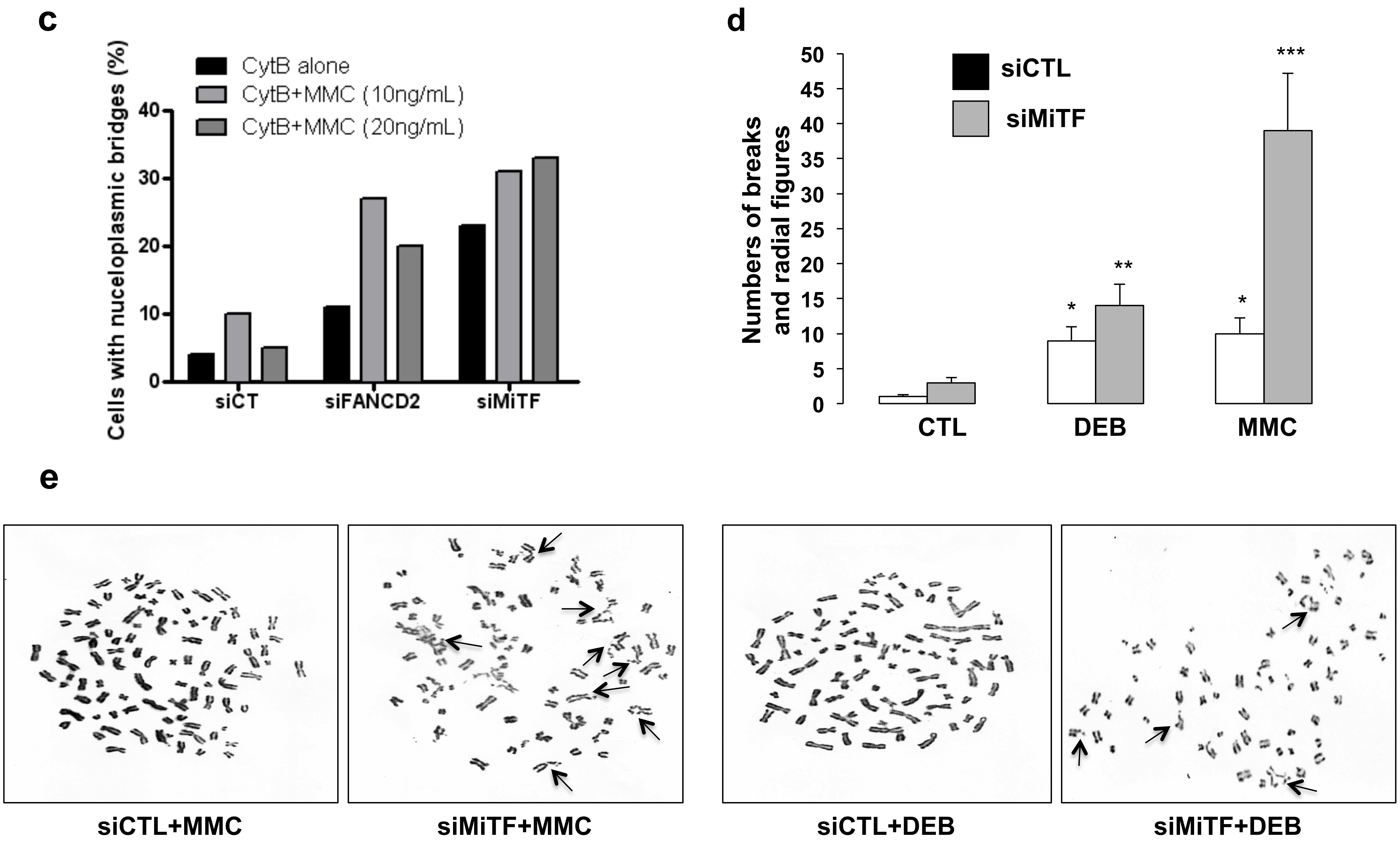

Supplemental Figure 2 Middle– Bourseguin et al.

f

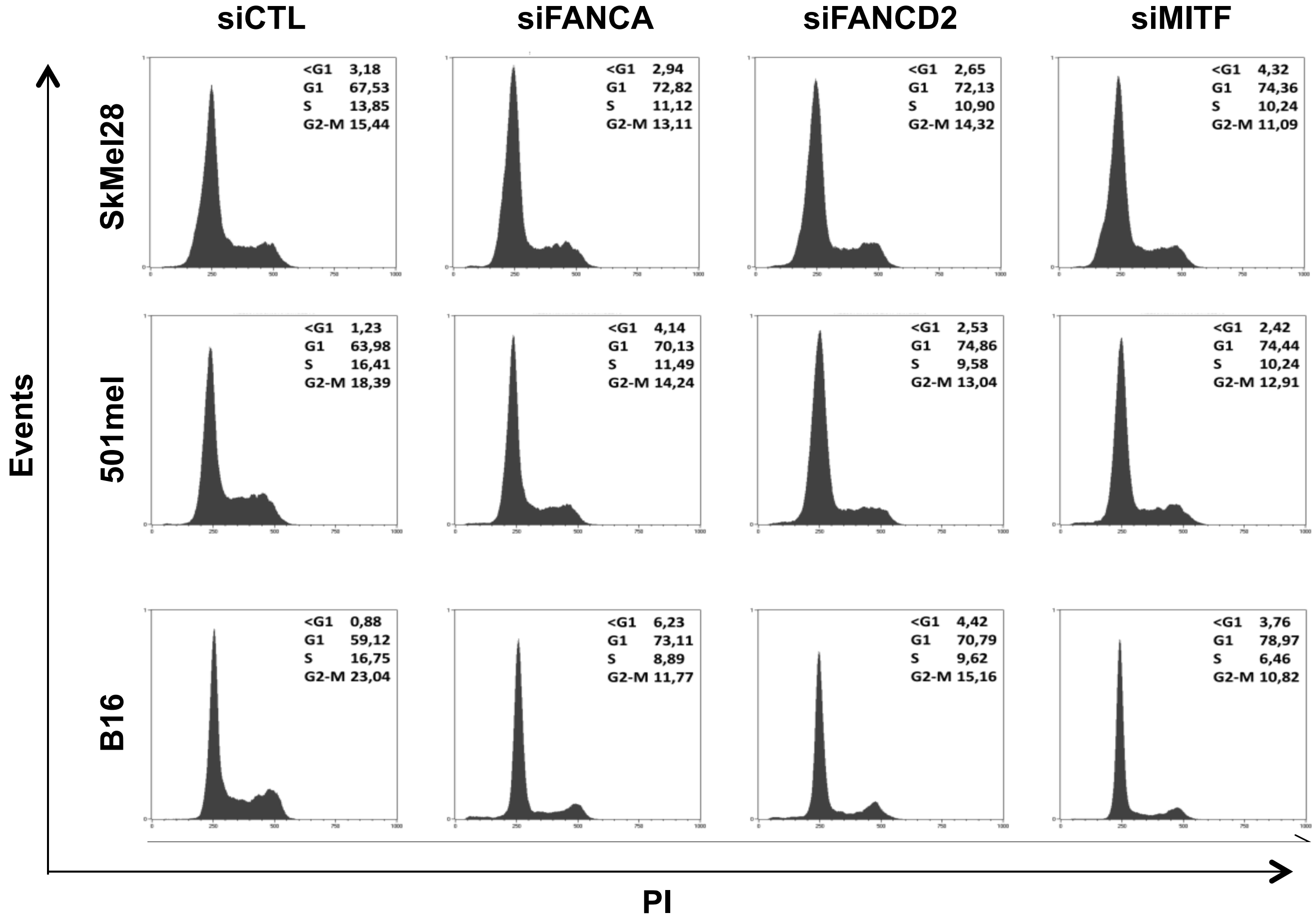

g

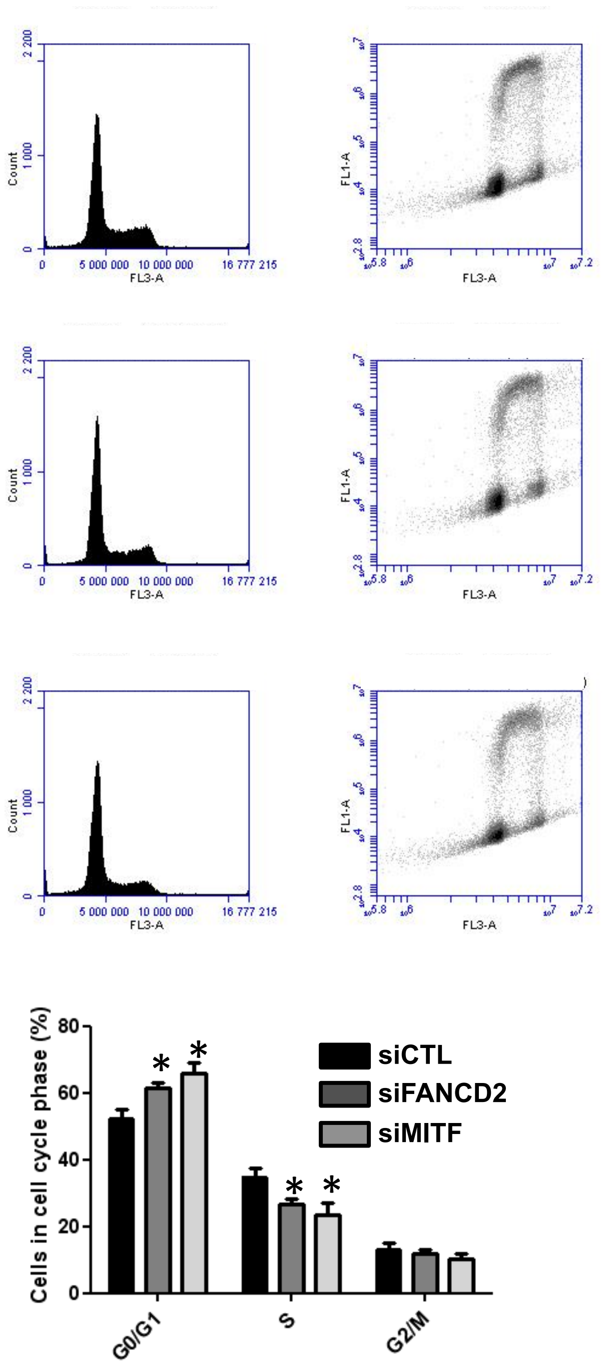

Supplemental Figure 2 Bottom – Bourseguin et al.

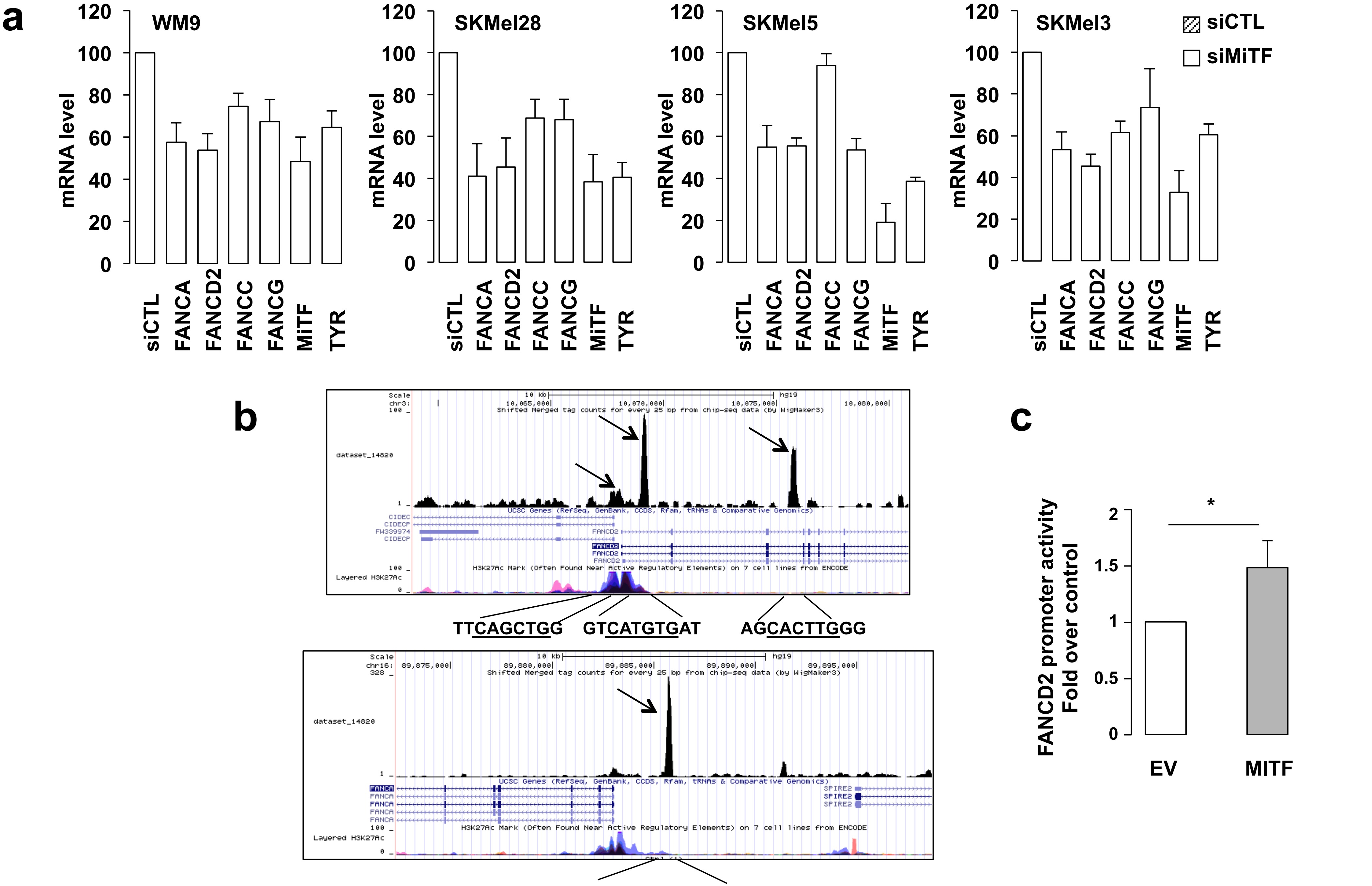

Supplemental Figure 3 Top – Bourseguin et al.

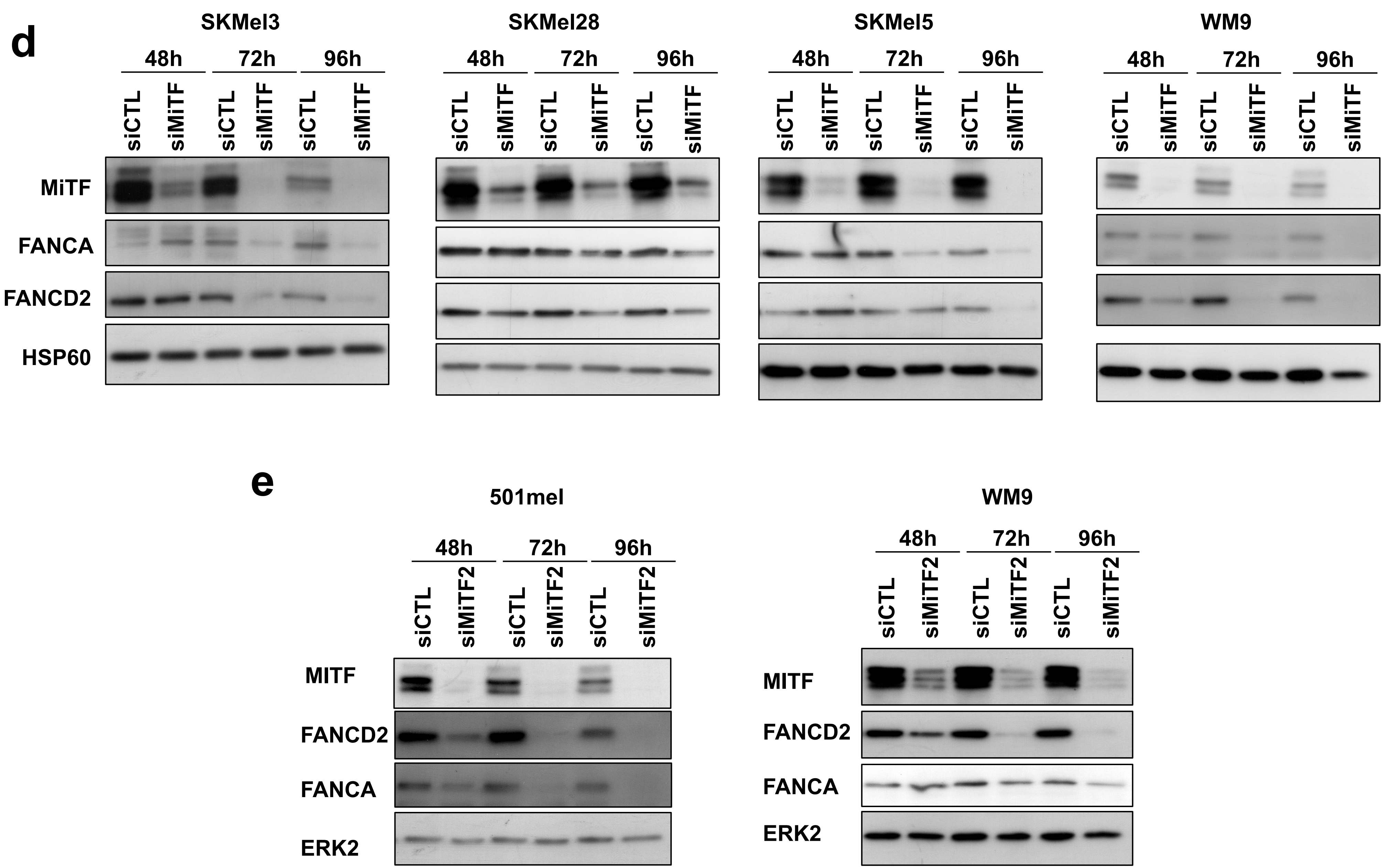

Supplemental Figure 3 Bottom – Bourseguin et al.

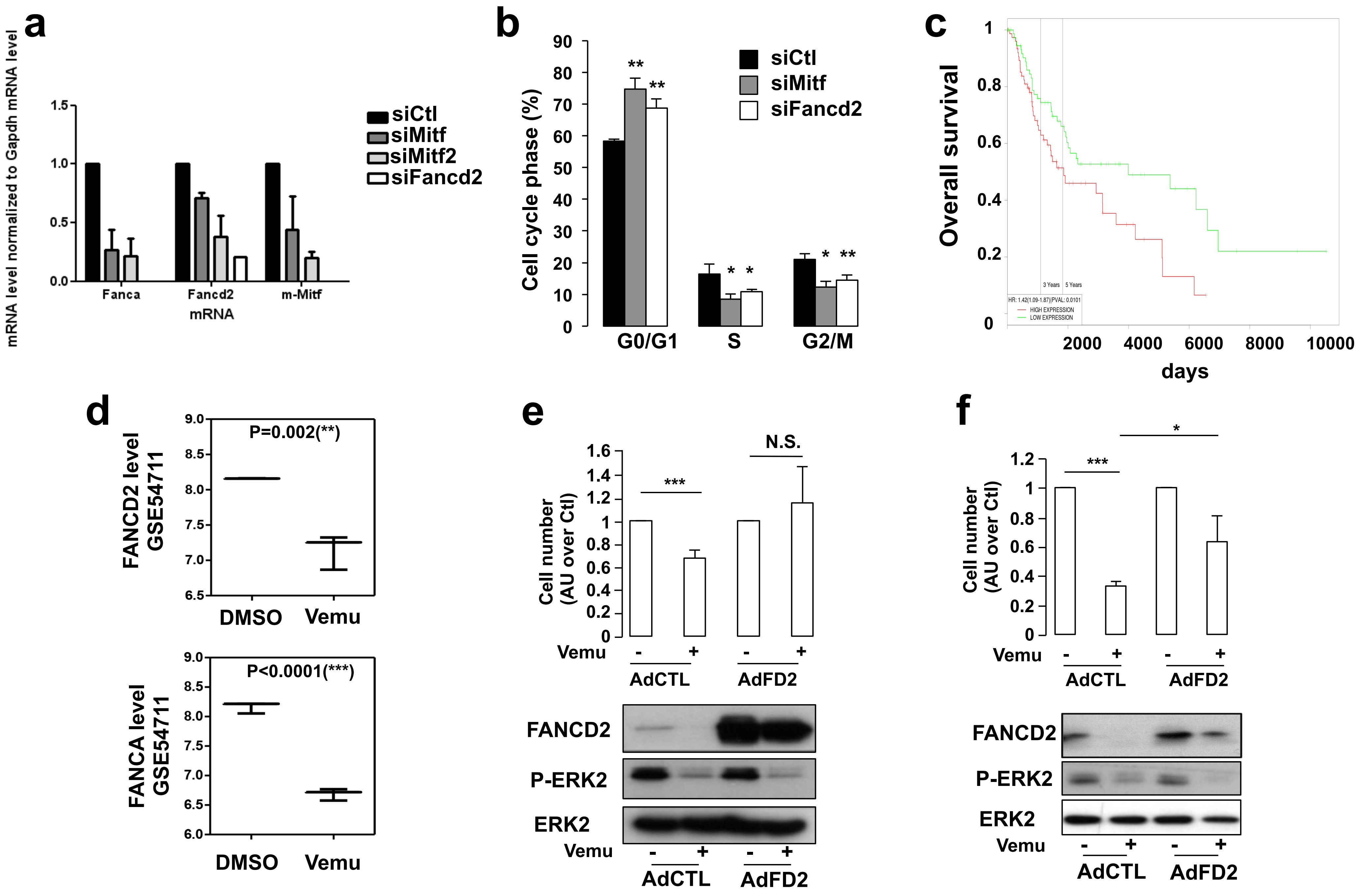

Supplemental Figure 4 – Bourseguin et al.
